# Wnt3a expression is associated with epithelial-mesenchymal transition and promotes colon cancer progression

**DOI:** 10.1186/s13046-014-0107-4

**Published:** 2014-12-11

**Authors:** Lisha Qi, Baocun Sun, Zhiyong Liu, Runfen Cheng, Yixian Li, Xiulan Zhao

**Affiliations:** Department of Pathology, Tianjin Medical University Cancer Institute and Hospital, Tianjin, 300060 China; Department of Pathology, Tianjin Medical University, Tianjin, 300070 China; The Key Laboratory of Tianjin Cancer Prevention and Treatment, Tianjin, 300060 China; National Clinical Research Center for Cancer, Tianjin, 300060 China

**Keywords:** Wnt3a, Epithelial-mesenchymal transition, Wnt/β-catenin pathway, Colon cancer

## Abstract

**Introduction:**

Epithelial–mesenchymal transition (EMT) contributes to the progression and metastasis of cancer cells and is associated with a more invasive phenotype of cancer. The Wnt/β-catenin signaling pathway is one of the major pathways involved in EMT regulation. Many studies provide evidence that β-catenin, the key regulator of the canonical Wnt signaling pathway, is important in regulating EMT in cancer. However, the roles of Wnt3a, the representative canonical Wnt ligand, in EMT and colon cancer progression have not yet been fully explored.

**Methods:**

The expression levels of Wnt3a and EMT-associated proteins (E-cadherin, vimentin, and β-catenin) were assessed by immunohistochemistry in human colon cancer tissues to evaluate the clinicopathological significance of Wnt3a, as well as the correlation between Wnt3a and EMT. We then upregulated Wnt3a expression in HCT116 colon cancer cells, established a nude mouse xenograft model, detected the expression of EMT and Wnt/β-catenin signaling-associated proteins, and observed invasion and clone-initiating abilities.

**Results:**

In 203 human colon cancer tissue samples, Wnt3a protein overexpression was related to colon cancer histological differentiation (*P* = 0.004), clinical stage (*P* = 0.008), presence of metastasis and recurrence (*P* = 0.036), and survival time (*P* = 0.007) of colon cancer patients. Wnt3a expression was notably concomitant with EMT immunohistochemical features, such as reduced expression of the epithelial marker E-cadherin (*P* = 0.012), increased expression of the mesenchymal marker vimentin (*P* = 0.002), and cytoplasmic distribution of β-catenin (*P* = 0.021). Results of in vitro and in vivo experiments showed that Wnt3a overexpression could alter cell morphology, regulate EMT-associated protein expression, and enhance clone-initiation and invasion. Dkk1 (antagonist of Wnt/β-catenin signaling) could also partially reverse the expression of EMT-associated proteins in Wnt3a-overexpressing cells.

**Conclusions:**

Wnt3a expression was associated with EMT and promoted colon cancer progression. The EMT-inducing effect was partially due to the stimulative effect of Wnt3a on the Wnt/β-catenin pathway.

## Introduction

Colorectal carcinoma is the third most common cause of cancer-related deaths worldwide [1]. Although colorectal cancer diagnosis and treatment have significantly advanced over the past two decades, the five-year survival rate remains below 50%. More than 50% of colorectal cancer cases metastasize to the lymph nodes, liver, and lungs [2]. Thus, knowledge on the treatment of this disease would advance if its metastasis mechanism is deeply understood.

Epithelial–mesenchymal transition (EMT) is an early phase of the malignant transformation of epithelial cells. In EMT, cells lose their polarities and contacts with neighboring cells and then acquire mesenchymal-like and motile phenotypes [3]. Tumor-cell EMT is considered a crucial event in cancer metastasis. Numerous factors can reportedly induce or mediate EMT, and these factors are commonly associated with carcinoma progression.

The Wnt signaling pathway is pivotal in embryogenesis and development and is also widely implicated in a number of human diseases [4-6]. Deregulation of the Wnt signaling pathway has been associated with senescence bypass [7], aberrant cell growth [8], and cancer [9]. Signaling through the Wnt pathway starts with Wnt ligands, which consist of more than 19 closely related but distinct secreted cysteine-rich glycoproteins [10]. By activating multiple intracellular signaling cascades, Wnts control various cellular functions, including proliferation, tissue homeostasis, stem cell maintenance, and cell fate decisions [11,12]. Several Wnts such as Wnt1, Wnt3a, and Wnt7a stimulate the β-catenin-dependent pathway, called canonical Wnt signaling [12]. Other Wnts such as Wnt4, Wnt5a, and Wnt11 may activate the protein kinase c, calcium-calmodulin kinase, or Jun NH (2)-terminal kinase pathway, called non-canonical Wnt signaling [13].

The canonical Wnt/β-catenin signaling pathway is well established in colorectal oncogenesis, with >85% of these malignancies harboring mutations along the pathway that lead to constitutive activation. Many studies provide evidence that the Wnt/β-catenin signaling pathway plays an important role in EMT regulation [14,15]. However, most investigations on the effects of the canonical Wnt pathway on EMT have focused on β-catenin rather than on Wnt ligands despite the role of Wnt proteins as major initiating factors in the Wnt/β-catenin pathway. Blavier et al. found that Wnt1 overexpression in murine mammary epithelial cells in vitro could promote EMT and cell proliferation [16]. Bo and Kanzawa have shown that increased Wnt5a provokes EMT of pancreatic cancer and gastric cancer cells [17,18]. Wu reported that Wnt3 activates the Wnt/β-catenin pathway and promotes EMT-like phenotypes in trastuzumab-resistant HER2-overexpressing breast cancer cells [19]. Bao et al. found that Wnt3a could promote the EMT, migration, and proliferation of human lens epithelial cells [20]. Meanwhile, the role of Wnt3a in colorectal cancer EMT has not been fully explored.

In this study, we evaluated the clinicopathological significance of Wnt3a and analyzed the correlation between Wnt3a expression and EMT immunohistochemical features in tissue specimens from 203 colon cancer patients. The effects of Wnt3a ectopic expression in the colon cancer cell line HCT116 on the expression of epithelial and mesenchymal markers and EMT transcription factors were studied. We also investigated cell proliferation and invasion in cell cultures with Wnt3a overexpression, as well as tumor growth and metastasis in a colon cancer xenograft model. We further treated Wnt3a-overexpressing cells with a Wnt/β-catenin pathway antagonist Dkk1-conditioned medium and detected the expression of EMT-related proteins to verify whether Wnt3a promoted EMT by activating the Wnt/β-catenin signaling pathway.

## Material and methods

### Clinical samples

Tissue samples of colon cancer were harvested from 203 patients who had undergone surgery for colon cancer in Tianjin Medical University Cancer Institute and Hospital (Tianjin, China) between January 2002 and December 2004. None of the patients had received any chemotherapy or radiotherapy before their operation. Data of clinicopathological parameters were obtained from patients’ clinical records and pathological reports.

### Cell culture reagents and animals

The human colon cancer cell line HCT116 was obtained from the Cell Resource Center at the Institute of Basic Medical Sciences, Chinese Academy of Medical Sciences/Peking Union Medical College (Beijing, China). Cells were cultured in Iscove modified Dulbecco medium with 10% FBS. Dkk1 recombinant protein was obtained from R&D Systems. For Dkk1 administration in vitro, recombinant Dkk1 (1 μg/mL) was added to culture medium. Typically, 50% of the medium was replaced every 24 h with fresh conditioned medium containing Dkk1 at the original concentrations. Cells were harvested 48 h post-treatment, and total cell lysates were collected for measurement by Western blot. The micro-Boyden chambers used were from NeuroProbe (Gaithersburg, MD, USA). Antibodies to β-catenin, goat anti-rabbit, and goat anti-mouse IgG-FITC were from Santa Cruz Biotechnology (Santa Cruz, CA, USA). Antibodies to Wnt3a, Snail, Slug, and Twist were from Abcam (Cambridge, UK). Antibody to E-cadherin was from BD Biosciences (San Jose, CA, USA). Antibody to vimentin was from Epitomics (Burlingame, CA, USA). Phalloidin was from Invitrogen (Carlsbad, CA, USA). Alexa Fluor 488 and 546 were from Molecular Probes (Eugene, OR, USA). BALB/C nude mice (4–5 weeks old) were obtained from Wei Tong Li Hua Experimental Animal Company (Beijing, China).

### Immunohistochemical staining

Streptavidin–biotin–peroxidase staining was performed as previously described [21]. In a typical procedure, the sections were pretreated with microwaves, blocked, and incubated with a series of antibodies overnight at 4°C. Then, they were immunostained with HRP-conjugated antibody and signals were revealed using 3,3-diaminobenzidine buffer as substrate. In place of primary antibodies for the negative control, PBS was used.

The expression of Wnt3a, E-cadherin, vimentin, and β-catenin was analyzed only histologically in normal and neoplastic epithelial cells and not in stromal tissues. Wnt3a staining was considered immunoreactive when brown granules were identified in the cytoplasm. The staining intensity of Wnt3a was graded on a scale from 0 to 2 (0 for no staining, 1 for weak immunoreactivity, 2 for strong immunoreactivity). Percentage immunoreactivity was scored on a scale from 0 to 3 (0 for no positive cells, 1 for <25% of cells being positive, 2 for 25% to 50% of cells being positive, and 3 for >50% of cells being positive). We multiplied the two scores to obtain a composite Wnt3a expression score. Wnt3a expression was classified as negative (score = 0), weakly positive (score = 1, 2, or 3), or strongly positive (score = 4, 5, or 6). E-cadherin expression was considered to be positive if >90% of cancer cells exhibited a staining pattern similar to that in normal epithelial cells. Vimentin expression was classified as positive when >10% cells were stained. β-Catenin nuclear staining was considered positive if >10% of cells showed brown granules in nuclei.

### Plasmid transfection

Transfection with plasmid carrying Wnt3a and controlled scrambled plasmid (Genechem, Shanghai, China) was performed with Lipofectamine 2000 (Invitrogen, Carlsbad, CA, USA) according to the manufacturer’s instructions. To establish stable HCT116 cells that overexpressed Wnt3a, G418-resistant cells were screened.

### Western blot analysis

Protein (30–50 μg/lane) was separated by 10% SDS-PAGE and transferred to polyvinylidene difluoride membranes. Blots were blocked and incubated with primary antibodies overnight at 4°C, incubated with secondary antibody, and detected with ECL Western blot substrate (Millipore) according to the manufacturer’s instructions.

### Soft agar colony formation assay

To form bottom agar, 1.5 mL of culture medium containing 0.6% agarose was added to each 35 mm dish. Then, 1 mL of culture medium containing 0.6% agarose and 1 × 10^4^ cells were mixed gently at 37°C and plated onto the bottom agar. Dishes were incubated at 37°C and 5% CO_2_. Six days later, colonies (>50 μm) were counted from 10 random fields per dish.

### Matrigel invasion assay

Matrigel (BD Biosciences) with a final concentration of 1.5 mg/mL was added to the upper surface of the chamber filter (8 μm pore). Then, 200 μL of cell suspension (5 × 10^5^ cells/mL) contained in serum-free medium was added to the upper chamber, and 300 μL of culture medium supplemented with 20% FBS was added to the lower chamber. After incubation for 20 h, the passed cells were fixed and stained.

### Immunofluorescence confocal microscopy

Cells were cultured on sterile glass cover slips on the day before staining. Cells were fixed with 4% paraformaldehyde, quenched with 50 mmol/L NH_4_Cl, permeabilized in 0.2% Triton X-100, and blocked in 3% BSA. The slips were incubated with the primary antibodies overnight at 4°C, labeled with the specific secondary antibodies for 1 h in the dark, mounted, and visualized with a confocal laser scanning microscopy (Leica TCS SP5, Leica Microsystems).

### In vivo assay

Twenty mice were randomly and evenly divided into two groups and given either 3 × 10^6^ control or HCT116 cells overexpressing Wnt3a (clone7) by subcutaneous injection in right groin. Tumor size was measured every 3 days for 21 days. Tumor volumes were calculated using the following formula: volume = [length(in millimeters) × width^2^[in square millimeters])/2. Tumor samples were formalin fixed and paraffin embedded.

### Statistical analysis

SPSS v.16.0 software (SPSS Inc., Chicago, IL, USA) was used for data analysis. The associations between Wnt3a and clinicopathologic parameters and the differential expression of E-cadherin, vimentin and β-catenin between different Wnt3a expression level groups were assessed with Fisher’s exact test and chi-square test. Differences or correlations between groups were assessed by the Mann–Whitney U-test, Student’s t-test and Pearson’s correlation test. Survival analysis was carried out according to Kaplan–Meier. Differences in survival curves were assessed using the log rank test. Significance was set at *P* < 0.05.

## Results

### Wnt3a expression is increased in colon carcinomas and correlates with the clinical outcome of patients

**Figure 1:**
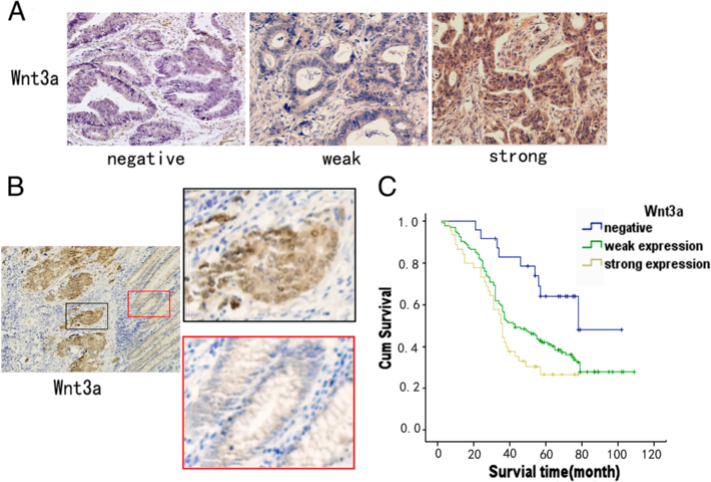
**Expression of Wnt3a by immunohistochemical staining in human colon cancer tissue samples. (A)** Representative colon cancer samples with Wnt3a negative (left), weak (middle), and strong (right) expression, 400×. **(B)** Strong expression of Wnt3a was observed in invasive front, 200×. **(C)** Kaplan–Meier survival analysis showing that Wnt3a-negative patients had longer survival time than Wnt3a-weak expression or Wnt3a-strong expression patients.

Among 203 samples, 179 (88.2%) showed positive Wnt3a expression, whereas the remaining 24 (11.8%) were negative. Tumors were categorized as strong expression, weak expression, or negative for Wnt3a (Figure 1A). Relationships between Wnt3a expression levels in colon cancer and each clinicopathological parameter were analyzed Table 1). Wnt3a expression level in colon cancer was found to increase with decreased differentiation grade. Wnt3a was strongly expressed in samples with higher clinical stages and metastasis/recurrence. Wnt3a expression was not significantly correlated with gender, age, and tumor size. Differences in Wnt3a expression level within tumors were found, with strong Wnt3a expression observed in tumor cells located close to stroma, implicating that Wnt3a may be involved in tumor progression in colon cancer (Figure 1B).

**Table 1:** **Correlation between Wnt3a and clinicopathologic characteristics of patients with colon cancer**

**Viariant**	**Total**	**Wnt3a expression**			**χ** ^**2**^	***P*** **Value**
		**Negative (%)**	**Weak expression (%)**	**Strong expression (%)**		
Age						
<45	27	2 (7.4)	17 (63)	8 (29.6)	1.340	0.512
≥45	176	22 (12.5)	117 (66.5)	37 (21.0)		
Sex						
Male	95	10 (10.5)	68 (71.6)	17 (17.9)	2.563	0.278
Female	108	14 (13)	66 (61.1)	28 (25.9)		
Tumor size						
≥5 cm	166	6 (9.1)	41 (62.1)	19 (28.8)	2.775	0.250
<5 cm	137	18 (13.1)	93 (67.9)	26 (19.0)		
Histological differentiation						
Well differentiated	16	5 (31.2)	10 (62.5)	1 (6.2)	15.127	0.004*
Moderately differentiated	101	11 (10.9)	74 (73.3)	16 (15.8)		
Poorly differantiated	86	8 (9.3)	50 (58.1)	28 (32.6)		
Clinical stage						
TNMI	10	5 (50.0)	4 (40.0)	1 (10.0)	17.456	0.008*
TNMII	128	15 (11.7)	86 (67.2)	27 (21.1)		
TNMIII	54	4 (7.4)	37 (68.5)	13 (24.1)		
TNMIV	11	0 (0.0)	7 (63.6)	4 (36.4)		
Metastasis/recurrence						
Absent	135	20 (14.8)	91 (67.4)	24 (17.8)	6.674	0.036*
Present	68	4 (5.9)	43 (63.2)	21 (30.9)		

*Significantly different.

Furthermore, Kaplan–Meier survival analysis showed that the total survival time for patients in the Wnt3a-negative group was significantly longer than for those in the Wnt3a-weak expression or Wnt3a-strong expression group (*P* = 0.007). The average survival time for Wnt3a-negative patients was 76.4 months, whereas the average survival time for Wnt3a-weak expression and Wnt3a-strong expression group patients were 57.6 and 41.7 months respectively (Figure 1C).

### Wnt3a expression was concomitant with EMT immunohistochemical features

**Figure 2:**
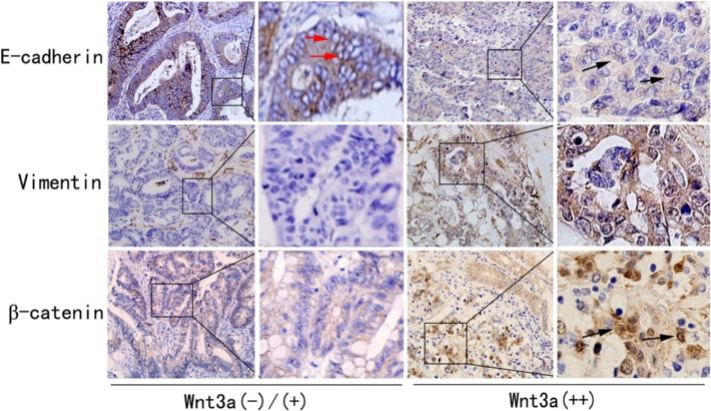
**Wnt3a expression was concomitant with EMT immunohistochemical features in human colon cancer tissue samples.** E-cadherin expression was higher in Wnt3a negative (−) or weak expression (+) colon cancer tissue sections than in strong-expression (++) samples. E-cadherin was mainly localized in membrane of Wnt3a (−)/(+) samples (red arrows) and in cytoplasm of Wnt3a (++) samples (black arrows). Tumor cells in Wnt3a (−)/(+) section did not express vimentin, whereas several tumor cells in Wnt3a (++) section showed expression of vimentin. In Wnt3a (−)/(+) sections, tumor cells displayed weak and only membrane-localized β-catenin expression, whereas tumor cells in Wnt3a (++) section showed nuclear β-catenin accumulation (black arrows), 400×.

To assess the relationship between Wnt3a and EMT in colon cancer, we investigated the expression of the EMT-associated markers E-cadherin, vimentin, and β-catenin (also a marker of Wnt/β-catenin pathway activation). As shown in Table 2 and Figure 2, the Wnt3a-negative group showed higher E-cadherin expression and lower vimentin and nuclear β-catenin expression than the positive group. β-Catenin was mainly expressed in cytoplasm in the Wnt3a-negative group and in the nucleus in the positive group. The expression of Wnt3a correlated with the expression of E-cadherin (*r* = −0.208, *P* < 0.05), vimentin (*r* = 0.247, *P* < 0.001), and β-catenin (nuclear) (*r* = 0.194, *P* < 0.05). These data provided proof about the role of Wnt3a as a potent activator of Wnt/β-catenin signaling and as a regulator involved in tumor progression in colon cancer.

**Table 2:** **Correlation between expression of Wnt3a and EMT-associated proteins**

**Variant**	**Total**	**Wnt3a expression**			**χ** ^**2**^	***P*** **Value**
		**Negative (%)**	**Weak expression (%)**	**Strong expression (%)**		
E-cadherin expression						
Negative	16	0 (0.0)	8 (50.0)	8 (50.0)	8.798	0.012*
Positive	187	24 (12.8)	126 (67.4)	37 (19.8)		
Vimentin expression						
Negative	187	24 (12.8)	127 (67.9)	36 (19.3)	12.459	0.002*
Positive	16	0 (0.0)	7 (43.8)	9 (56.2)		
β-catenin nuclear expression						
Negative	167	22 (13.2)	114 (68.3)	31 (18.6)	7.698	0.021*
Positive	36	2 (5.6)	20 (55.6)	14 (38.9)		

*Significantly different.

### Wnt3a overexpression induced mesenchymal phenotype and increased expression of Snail in HCT116 cells

**Figure 3:**
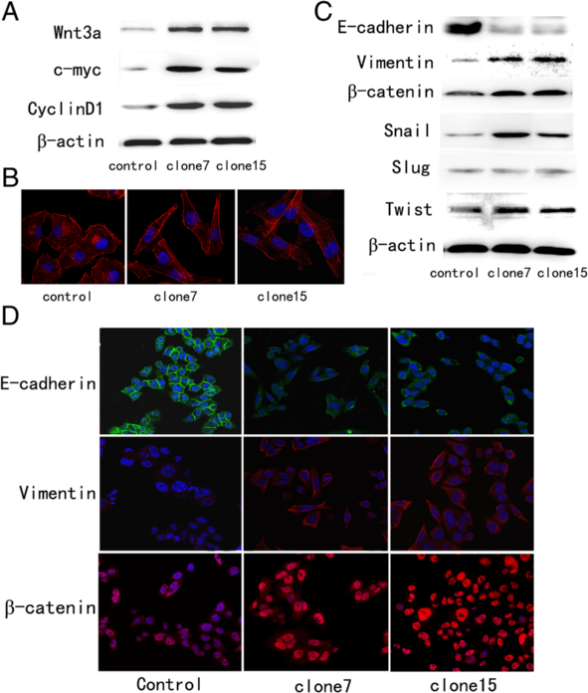
**Wnt3a overexpression induced mesenchymal phenotype and increased expression of mesenchymal markers. (A)** Wnt3a protein levels were significantly increased in clone7 and clone15 HCT116 cell pools transfected with Wnt3a plasmid. Then, c-myc and CyclinD1 protein expression increased in clone7 and clone15 HCT116 cell. **(B)** Immunofluorescent staining of F-actin. Wnt3a-overexpressing cells exhibited dramatic changes in cell morphology from a tight packed, polarized, and epithelial-like appearance to a scattered, irregular, and fibroblastic-like shape. **(C)** Wnt3a-overexpressing cells showed lower E-cadherin expression but higher vimentin expression. β-Catenin expression did not markedly change in three groups of cells. EMT regulatory proteins including Snail, Slug, and Twist were detected. Snail was upregulated in Wnt3a-overexpressing cells compared with control cells, whereas Slug and Twist expression did not significantly change. **(D)** Immunofluorescent staining of E-cadherin, vimentin, and β-catenin. Wnt3a-overexpressing cells showed lower E-cadherin expression and higher vimentin expression. More β-catenin accumulation in nucleus was observed in Wnt3a-overexpressing cells than in control cells. A green or red signal represents staining for corresponding protein, whereas a blue signal represents nuclear DNA staining by 4′,6-diamidino-2-phenylindole.

We established stable Wnt3a-overexpressed colon cancer cells to study the EMT-promoting effect of Wnt3a on colorectal cancer cells. To rule out clone-to-clone variations, we selected two clones (clone7 and clone15). HCT116 cells with Wnt3a overexpression had increased expression of c-myc and cyclin D1 (Figure 3A), which are the best-known target proteins of canonical Wnt signaling [19,20], thereby confirming activation of the signaling pathway.

EMT is a multistep process in which cells undergo molecular alterations that facilitate dysfunctional cell–cell adhesive interactions and reorganization of cytoskeleton, resulting in loss of apical polarity and acquisition of a more spindle-shaped morphology. Thus, we used phalloidin to dye fibrous actin (F-actin), a representative of cytoskeleton, and observed that Wnt3a overexpression caused HCT116 cells to form structures with irregular shape and non-uniform composition or density (Figure 3B). Western blot and immunofluorescence assays demonstrated that cells overexpressing Wnt3a had lower expression of E-cadherin and higher expression of vimentin than control cells (Figure 3C and 3D). In addition to classical EMT markers, we examined the expression of the EMT transcription factors Snail, Slug, and Twist. These markers could repress E-cadherin expression by direct binding to the E-boxes of the E-cadherin promoter. Among them, Snail was upregulated in cells overexpressing Wnt3a compared with control cells, whereas the expression of Slug and Twist did not significantly change (Figure 3C). Moreover, although total β-catenin expression did not markedly change in Western blot detection, immunofluorescence showed that more β-catenin accumulated in the nucleus of cells overexpressing Wnt3a than in that of control cells (Figure 3D). All these findings suggested that cells overexpressing Wnt3a were more predisposed to mesenchymal differentiation.

### Wnt3a promotes *in vitro* clone-initiating and invasion abilities, *in vivo* tumor growth and metastasis of HCT116 cells

**Figure 4:**
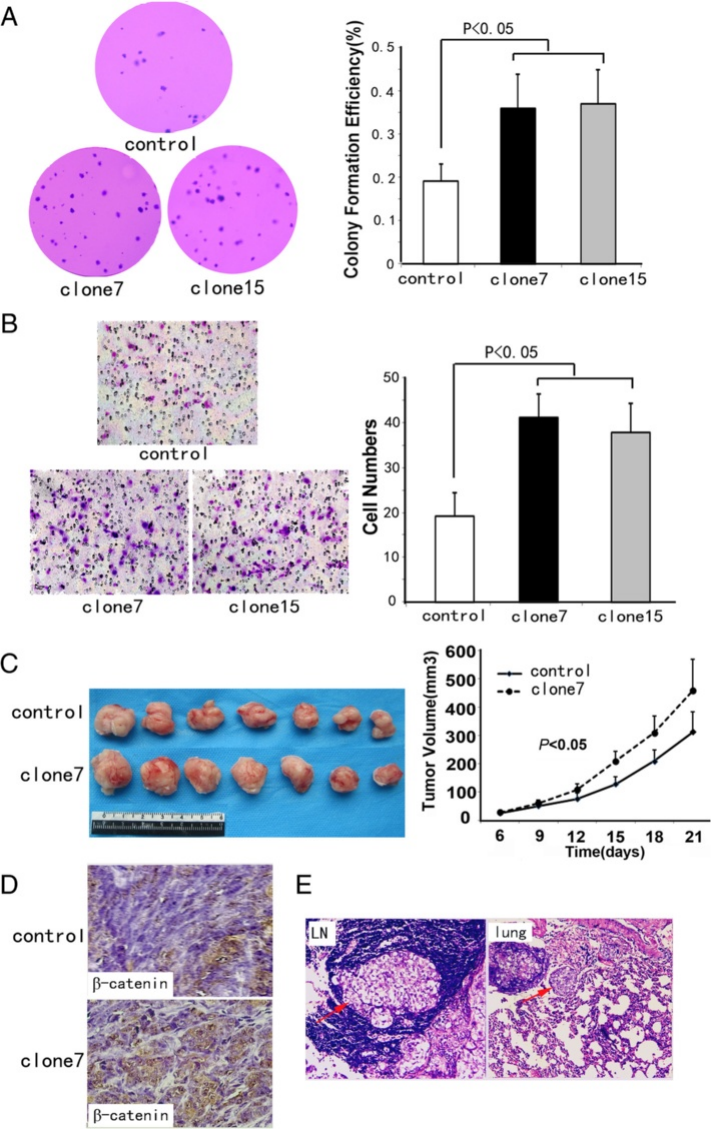
**Effect of Wnt3a overexpression on in vitro clone-initiation and invasion abilities and in vivo tumor growth and metastasis. (A)** Wnt3a overexpression promoted HCT116 anchorage-independent growth in soft agar. Colonies in soft agar culture were stained (left), 200×. Histogram showing colony formation efficiency (right). **(B)** Cells invading through matrigel-coated transwell inserts were stained (left), 200×. Invading cells were counted in five pre-determined fields (right), 400×. **(C)** Photograph of representative tumors from mice injected with control or Wnt3a transfected HCT116 cells (clone7) (left). Wnt3a-overexpressing cells produced larger tumor masses than control cells (right). **(D)** Immunohistochemical staining of β-catenin expression in harvested mouse tumor samples. Tumors overexpressing Wnt3a (clone7) exhibited increased nuclear β-catenin expression compared with control tumors, 400×. **(E)** Mice injected with Wnt3a-overexpressing cells (clone7) showed lymph node metastasis and lung metastasis (red arrows); hematoxylin and eosin staining, 200 × .

Anchorage-independent growth, one of the most important malignant features of cancer cell stemness, was found to be significantly increased in cells overexpressing Wnt3a (Figure 4A).

Compared with epithelial cells, mesenchymal cells generally defined cell polarity, cytoskeletal structures and cell-ECM interactions. Thus, the process of EMT can directly lead to increased invasive potential of tumor cells. As expected, more cells overexpressing Wnt3a invaded through the Matrigel than control cells (Figure 4B).

In agreement with *in vitro* findings, clone7 cells overexpressing Wnt3a grew into larger tumor masses than control cells (Figure 4C). To assess canonical Wnt signal activity in xenografts, we then performed β-catenin immunohistochemical staining on the sections of xenograft tissues. The nuclear expression of β-catenin significantly increased in Wnt3a tumors compared with control tumors (Figure 4D). Among the 10 mice injected with clone7 cells, one showed lung metastasis and one showed lymph node methastasis (Figure 4E). Meanwhile, among the 10 mice injected with control cells, no mouse showed lymph node or lung metastasis and only two showed tumor invasion into the surrounding fatty tissue.

### Dkk1 abolishes the expression of EMT-associated proteins in Wnt3a-overexpressing HCT116 cells

**Figure 5:**
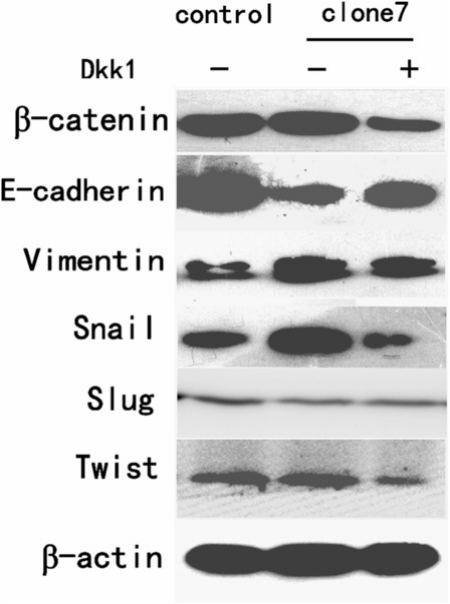
**Dkk1 disrupted the expression of EMT-associated proteins in Wnt3a overexpressing HCT116 cells.** After Dkk1 treatment, Wnt3a-overexpressing cells showed decreased β-catenin, vimentin, Snail, and Twist, expression but increased E-cadherin expression.

To verify whether the EMT promotion effect of Wnt3a was due to Wnt/β-catenin pathway activation, we utilized the Wnt/β-catenin pathway inhibitor Dkk1. Dkk1 functions as an antagonist of the Wnt/β-catenin pathway by binding to lipoprotein receptor-related protein 5 or 6 (LRP5/6) and preventing the formation of Wnt-Fz-LRP ternary complexes and the downstream signaling transduction. After Dkk1 treatment, Wnt3a-overexpressing cells showed decreased β-catenin expression, indicating the effectiveness of Dkk1 as an inhibitor of Wnt/β-catenin pathway. Western blot assays also demonstrated that Wnt3a-overexpressing cells treated with Dkk1 had higher expression of E-cadherin and lower expression of vimentin, Snail, and Twist compared with untreated cells (Figure 5). This finding indicated that Wnt/β-catenin pathway activation played an important role in the EMT-inducing effect of Wnt3a. Meanwhile, Dkk1 did not restore the expression of EMT-associated proteins in Wnt3a-overexpressing cells to the same level as the control cells, suggesting that Wnt3a may promote EMT through distinct mechanisms other than activating the Wnt/β-catenin pathway.

## Discussion

Wnt3a is a Wnt protein that activates the canonical Wnt pathway. Wnt3a stimulates tumor progression in glioblastoma [22], breast and prostate cancers [23,24], and malignant mesothelioma [25]. Other studies have shown that Wnt3a serves as a tumor suppressor based on two main findings. One is that bones engrafted with Wnt3a-expressing multiple myeloma H929 cells are preserved; the other is that treatment of myelomatous SCID mice carrying the primary disease with recombinant Wnt3a stimulates bone formation and attenuates multiple myeloma growth [26]. Marit et al. reported that Wnt3a inhibits the proliferation of several B-acute lymphoblastic leukemia cell lines [27]. In the present study, we initially analyzed Wnt3a expression in a large array of colon cancer tissue samples to determine its role in colon-cancer progression. We observed a significant correlation between Wnt3a expression and histological differentiation, clinical stages, metastasis, and recurrence, indicating that the upper stream factor of the Wnt signaling pathway may play an important role in colon-cancer progression. This result was consistent with a recent study on colorectal cancer, in which results reveal that Wnt3a is highly expressed in the primary and metastatic sites and is significantly associated with expression of the metastasis-related protein matrix metalloproteinase (MMP)-9 [28].

EMT is involved in numerous normal developmental processes and in cancer progression. EMT involves biochemical changes that result in decreased expression of the epithelial marker E-cadherin but increased expression of mesenchymal markers, such as vimentin. Cancer cells undergoing EMT are suitable for migration, invasion, and proliferation, thereby facilitating tumor progression. EMT involves different regulatory signaling pathways. Although the effects of Wnt/β-catenin signaling in promoting EMT during physiological or pathological processes have been extensively studied, the present study was the first to demonstrate the EMT-inducing ability of Wnt3a in colon cancer. In human colon cancer tissue samples, lower levels of E-cadherin expression, as well as higher levels of vimentin expression and β-catenin nuclear distribution, were observed in the Wnt3a strong group than in the weak and negative expression groups. This finding suggested that Wnt3a may contribute to EMT in colon cancer. EMT induction in colon cancer cells by Wnt3a was confirmed through the altered morphology and expression of the EMT-associated markers, as well as enhanced invasion capacities. Moreover, previous reports have indicated that cancer cells undergoing EMT share the properties of stem cell-like cells [29-31].

In the present study, we found that Wnt3a overexpression could promote colon-cancer cell growth in anchorage-independent growth in soft agar and increased the expression of c-myc, which is a major target molecule of transcription T-cell factor/lymphoid enhancer factor (TCF/LEF) promoter and also a cancer stem cell marker. We further demonstrated that Wnt3a could promote the expression of Snail, which is a key transcription EMT regulator. We also found that Wnt3a has significant stimulative effects on tumor growth and metastasis in nude mice. These findings demonstrated that ectopic Wnt3a expression exerted EMT-inducing effects that promote the progression of colon cancer. Meanwhile, another study on hepatocellular carcinoma showed that recombinant Wnt3a could not induce Huh-7 or HepG2 cells to undergo EMT in normoxia. Yoshida also indicated that oxidative stress could induce canonical Wnt activation. All these observations emphasize the importance of the microenvironment for Wnt signaling and EMT [32,33]. We suggested that the inconsistencies may be attributed to the different cell types and tissues.

β-Catenin has a dual cellular function and is involved in both transcription regulation and cell adhesion. When Wnt ligands such as Wnt1, Wnt3a, and Wnt7a act on their cell-surface receptors, cytoplasmic β-catenin is stabilized by its release from the axin complex and accumulation in the nucleus, where it binds to TCF/LEF and stimulates the expression of various genes involved in EMT [34]. β-Catenin has also been identified as a cadherin-binding protein. The nuclear translocation of β-catenin leads to the breakdown of cell-to-cell adhesion formed by β-catenin and E-cadherin. This phenomenon has been extensively studied in both morphological and biochemical EMT processes regardless of inducers and origins [35]. Our results showed that ectopic Wnt3a expression in HCT116 increased the expression and intracellular distribution of β-catenin and of the well-established target proteins of Wnt/β-catenin signaling, namely, c-myc, and cyclin D1. However, the inhibition of Wnt/β-catenin signaling by Dkk1 disrupted Wnt3a-induced EMT. All results indicated that the EMT-inducing effect of Wnt3a in colon cancer may be at least partially due to the stimulative effect of Wnt3a on the Wnt/β-catenin pathway.

After treating with Dkk1, the expression of EMT-associated proteins in Wnt3a-overexpressing cells did not recover to the same level as that of control cells. Thus, a Wnt3a-induced EMT mechanism that is independent of the Wnt/β-catenin pathway may exist. Another possibility was that the cancer cells may be responsive to Dkk1 differently in 2-D versus 3-D cultures, which more closely mimic the in vivo environment [36,37].

Multiple interactions were observed between Wnt3a and other families of signaling molecules, such as FAK, TGF-β, and EGF [38-41]. Nalesso reported that Wnt3a could activate both canonical and non-canonical Wnt pathways in the articular chondrocyte [42]. Sonderegger reported that Wnt3a could activate phosphatidylinositide3-kinase (PI3K)/AKT signaling, which could potentially cross-talk to canonical Wnt signaling and affect MMP-2 secretions to promote the invasion of human trophoblasts [43]. Studies designed to elucidate the interactions of Wnt3a with other EMT-associated signaling pathways in colon cancer are currently under way.

In conclusion, we showed that Wnt3a expression activated the Wnt/β-catenin signaling pathway, and this activation was a mechanism underlying EMT in colon cancer. The results provide better understanding of the importance of EMT in tumor development and may enable the establishment of clinically useful therapy targets.
